# Global trend analysis and risk evolution of asbestos-related ovarian cancer: a population-based study and future prediction (1990–2021)

**DOI:** 10.3389/fpubh.2025.1698477

**Published:** 2026-01-02

**Authors:** Xiaolong Li, Jiwei Li, Dongyong Shan, Ming Zhou, Mengna Li, Yinghua Li, Man Xia, Hongyu Deng

**Affiliations:** 1 Department of Clinical Laboratory, Hunan Key Laboratory of Oncotarget Gene, Hunan Cancer Hospital and The Affiliated Cancer Hospital of Xiangya School of Medicine, Central South University, Changsha, China; 2 Department of Oncology, The Second Xiangya Hospital, Central South University, Changsha, China; 3 Department of Gynecological Oncology, Hunan Cancer Hospital and the Affiliated Cancer Hospital of Xiangya School of Medicine, Central South University, Changsha, China

**Keywords:** asbestos, epidemiology, GBD, global disease burden 2021, ovarian cancer

## Abstract

Existing evidence demonstrates that asbestos exposure is an environmental risk factor for ovarian cancer (OC). Using comprehensive data from the 2021 Global Burden of Disease Study, this research quantified the epidemiological burden of asbestos-related ovarian cancer. In 2021, asbestos exposure accounted for 5,587 OC deaths—a 51.2% increase from 1990. However, the age-standardized mortality rate (ASMR) decreased from 0.11 (95% UI: 0.05–0.17) to 0.07 (95% UI: 0.03–0.11) per 100,000 people between 1990 and 2021. Similarly, the disability-adjusted life-year (DALY) rate declined from 1.93 (95% UI: 0.92–3.03) to 1.15 (95% UI: 0.56–1.85) per 100,000 people, reflecting a 40.4% reduction in total DALYs. Projections based on the Bayesian age-period-cohort (BAPC) model indicate declining incidence and mortality rates over the next 25 years. These findings suggest asbestos’ role as an independent OC risk factor is diminishing, warranting further investigation into its synergistic effects and co-pathogenesis with other etiological factors.

## Introduction

Ovarian cancer (OC), a molecularly diverse gynecologic malignancy, has demonstrated evolving epidemiological patterns linked to environmental carcinogen exposure. To understand the specific impact of environmental risks, it is crucial to first recognize the broader disease landscape. Recent comprehensive analyses of the Global Burden of Disease (GBD) study have highlighted that the global incidence, mortality, and disability-adjusted life years (DALYs) of ovarian cancer have shown a persistent upward trajectory from 1990 to 2021, driven largely by demographic shifts and epidemiological transitions (1). Against this backdrop of increasing global burden, identifying modifiable risk factors remains a priority. Accounting for 5% of female cancer mortality globally, OC exhibits marked geographical disparities in incidence rates, ranging from 4.59 ASMR in low-income regions to 13.22/100,000 in high-income countries. Despite therapeutic advancements, 5-year survival rates remain below 45%, necessitating urgent investigation into modifiable risk factors (2).

The carcinogenic effect of asbestos fibers may be mediated through multiple pathways: Firstly, asbestos fibers can enter the ovarian microenvironment through the reproductive tract via retrograde migration or be transferred to ovarian tissues through the abdominal lymphatic system. Their physical stimulation can trigger chronic inflammatory responses and induce the generation of reactive oxygen species (ROS), leading to DNA damage and genomic instability (3, 4). Secondly, animal experiments have shown that asbestos fibers can activate the NLRP3 inflammasome pathway, promoting the release of pro–inflammatory factors IL–1β and IL–18. This persistent inflammatory microenvironment not only accelerates epithelial–mesenchymal transition (EMT) but may also regulate the expression of oncogenes through epigenetic modifications (4–7). Moreover, the interaction between asbestos and the estrogen signaling pathway deserves attention. *In vitro* studies have confirmed that asbestos fibers can enhance the transcriptional activity of estrogen receptor *α* (ERα), thereby promoting the abnormal proliferation of ovarian epithelial cells (8).

This epidemiological study quantified the spatio–temporal evolution of the burden of ovarian cancer (OC) attributable to asbestos from 1990 to 2021, using advanced decomposition analysis methods to distinguish the effects of population aging, population growth, and risk exposure. By conducting comparative assessments of regions stratified by the Socio–Demographic Index (SDI) and performing predictive modeling up to 2045, we aim to identify critical intervention windows and provide a basis for the global occupational health protection policy framework.

## Materials and methods

### Data source

The dataset used in this study was obtained from the Global Health Data Exchange (GHDx; https://ghdx.healthdata.org), which is a comprehensive resource for the Global Burden of Disease Study 2021 (GBD 2021). Ovarian cancer (OC) cases were systematically classified according to the International Classification of Diseases, 10th Revision (ICD–10) code C56. Key indicators for assessing disease burden included incidence, DALYs, ASMR, ASDR, and estimated annual percentage change (EAPCs). The methodological framework used in the GBD analysis has been elaborated in previous epidemiological studies (9–11). Our analysis was grounded in the Comparative Risk Assessment (CRA) framework of the GBD 2021 study (12). Within this framework, the association between occupational asbestos exposure and ovarian cancer is a pre-defined exposure-outcome pair. The attributable disease burden was calculated using the Population Attributable Fraction (PAF), which was derived by GBD using Levin’s formula (12). Exposure estimates were obtained from GBD’s integrated exposure modeling, which treats occupational asbestos as a distinct risk factor, separate from other occupational carcinogens or environmental exposures. Consequently, the PAF for asbestos was computed independently based on its specific exposure distribution and relative risk, ensuring that the reported burden of ovarian cancer (in deaths and DALYs) is uniquely attributable to occupational asbestos exposure and not conflated with other risk factors.

The Socio–Demographic Index (SDI), a comprehensive indicator that combines per capita income and education level, is divided into five tiers to assess regional socioeconomic differences: low (<0.46), low–middle (0.46–0.60), middle (0.61–0.69), high-middle (0.70–0.81), and high (>0.81). The study developed predictive models for the age-standardized death rate (ASDR) and age-standardized incidence rate (ASIR) of ovarian cancer among different age groups from 2022 to 2046. This study adhered to the ethical principles of the Declaration of Helsinki and was approved by the Institutional Review Board of Hunan Cancer Hospital and the Second Xiangya Hospital of Central South University in Changsha, China.

### Disease prediction

ASDR and ASMR were used to evaluate the ovarian cancer disease patterns in different geographical regions while adjusting for differences in the population age distribution. Compared with conventional methods such as the generalized additive model and Poisson regression analysis, the Bayesian age-period-cohort (BAPC) modeling framework showed higher accuracy in predicting cancer epidemiological indicators. In this study, by combining the BAPC method with the nested Laplace approximation algorithm, the predicted data of cancer-related ASMR and ASDR from 2022 to 2046 were generated.

## Results

### The global incidence and mortality of ovarian cancer associated with asbestos exposure

**Figure 1:**
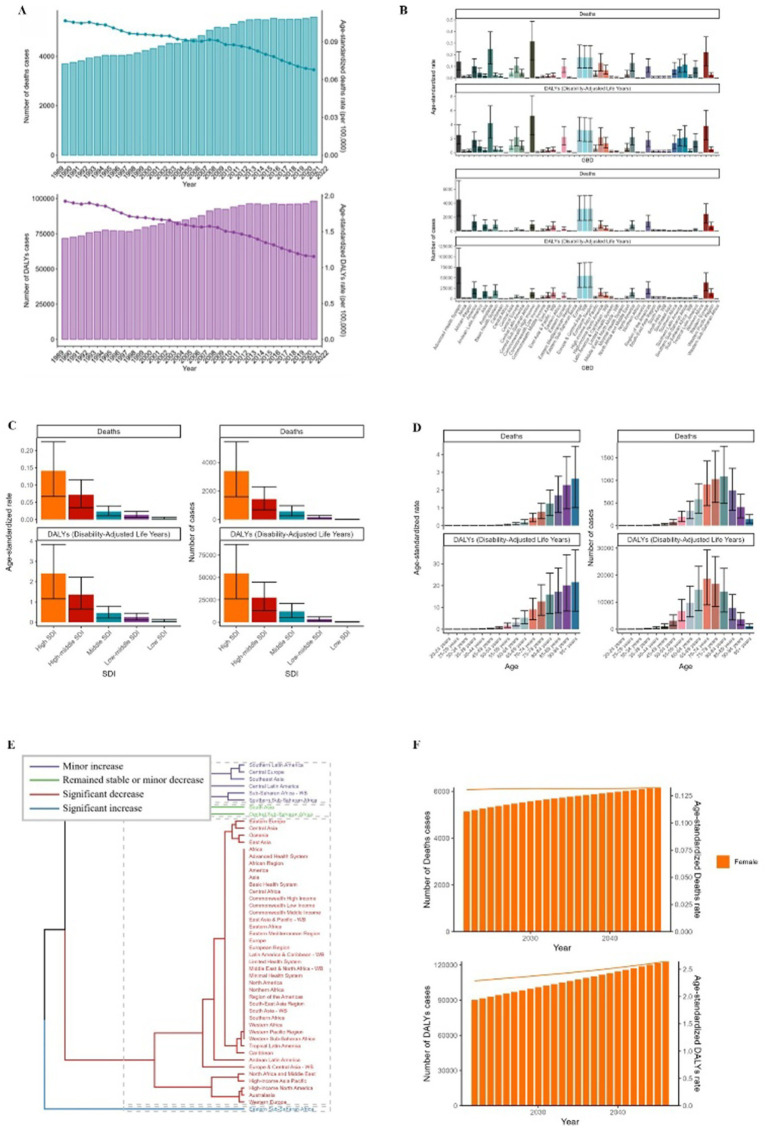
ASMR and ASDR of OC. **(A)** Numbers and age-standardized rates of asbestos-related deaths and DALYs from 1990 to 2021. **(B–E)** Numbers and age-standardized rates of asbestos-related deaths and DALYs in 2021 for different region **(B)**, SDI region **(C)**, different age groups **(D)** and different regions **(E)** in 2021. **(F)** The predicted results in asbestos-related numbers and age-standardized rates of deaths and DALYs globally from 1990 to 2046 of the BAPC model.

**Figure 2:**
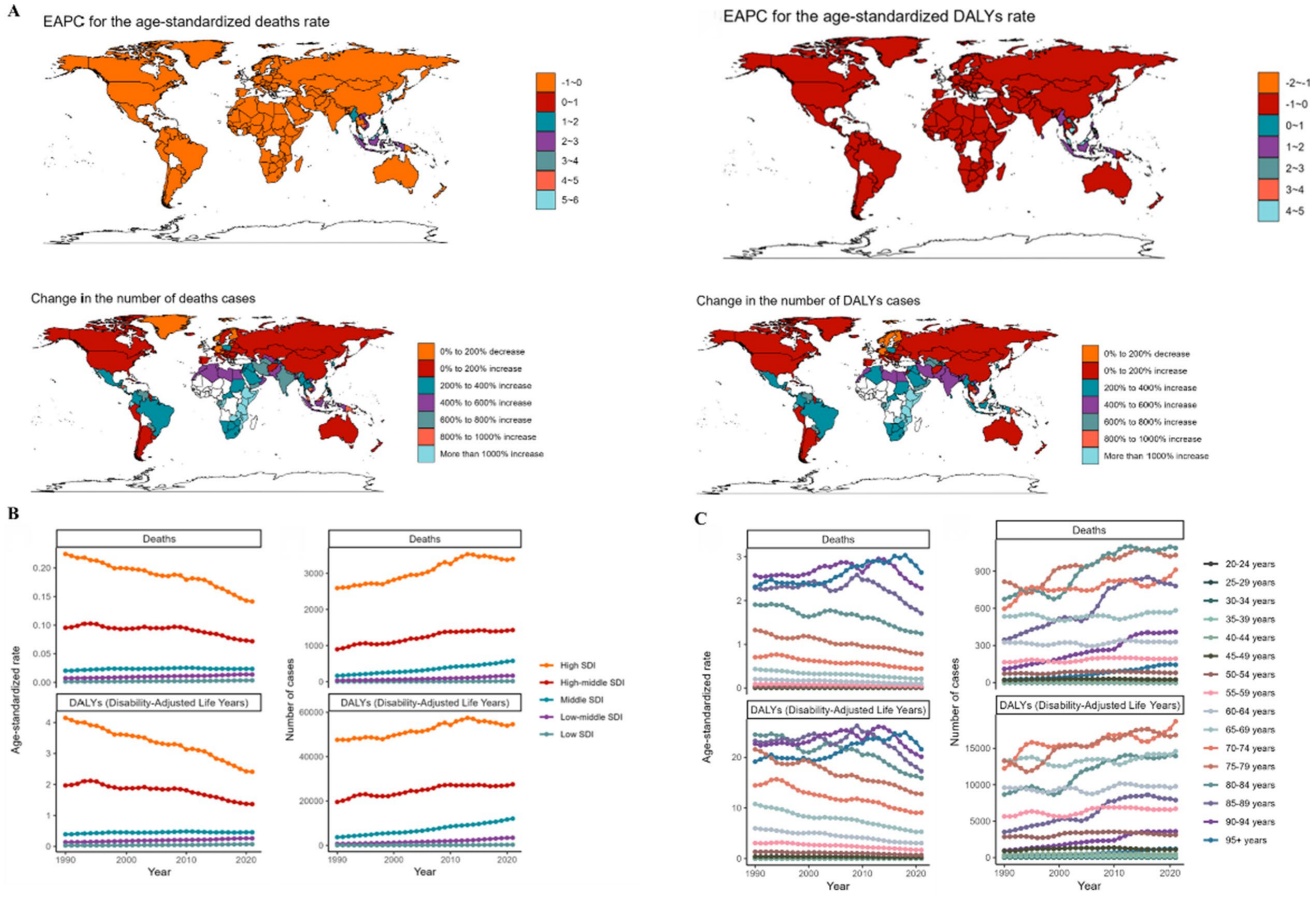
Change of the ASMR and ASDR of OC from 1990 to 2021. **(A)** EAPC for the age-standardized rates of asbestos-related deaths and DALYs in 1990 and 2021. **(B,C)** The trend of the numbers and age-standardized rates of asbestos-related deaths and DALYs from 1990 to 2021 for SDI region **(B)**, and different age groups **(C)**.

Since 1990, the global burden of ovarian cancer, assessed through mortality and DALYs, has risen persistently (Figures 1A, 2A). Deaths attributable to asbestos exposure in ovarian cancer increased substantially, from 3,694 (95% UI: 1,778–5,799) in 1990 to 5,587 (95% UI: 2,714–9,036) in 2021 (Figure 1A; Table 1). Corresponding asbestos-related DALYs also increased, from 71,726 (95% UI: 34,302–112,279) to 98,122 (95% UI: 47,184–156,828) by 2021 (Figure 1A; Table 2). In 2021, Europe reported the highest asbestos-related ovarian cancer mortality (3,197 deaths; 95% UI: 1,542–5,057) and DALYs (54,438; 95% UI: 27,135–85,151) among the 50 GBD regions (Figure 1B).

**Table 1:** The number of deaths cases and the age-standardized deaths rate attributable to asbestos exposure in 1990 and 2021, and its trends from 1990 to 2021 globally in ovarian cancer.

Characteristics	1990		2021		1990–2021
	Number of deaths cases (95% UI)	The age-standardized deaths rate/100000 (95% UI)	Number of deaths cases (95% UI)	The age-standardized deaths rate/100000 (95% UI)	EAPC (95% CI)
Global	3,694 (1778–5,799)	0.11 (0.05–0.17)	5,587 (2714–9,036)	0.07 (0.03–0.11)	−1.32 (−1.46 to −1.19)
Age					
20–24 years	0 (0–0)	0 (0–0)	0 (0–0)	0 (0–0)	−0.68 (−1.62–0.28)
25–29 years	0 (0–0)	0 (0–0)	0 (0–0)	0 (0–0)	−1.42 (−2.09 to −0.74)
30–34 years	0 (0–1)	0 (0–0)	0 (0–1)	0 (0–0)	−1.07 (−1.57 to −0.55)
35–39 years	2 (1–4)	0 (0–0)	3 (1–5)	0 (0–0)	−1.72 (−1.96 to −1.47)
40–44 years	8 (3–13)	0 (0–0)	10 (4–18)	0 (0–0)	−1.29 (−1.46 to −1.12)
45–49 years	20 (9–35)	0.01 (0–0.02)	25 (11–45)	0.01 (0–0.01)	−1.98 (−2.27 to −1.7)
50–54 years	73 (33–118)	0.03 (0.02–0.06)	80 (35–135)	0.02 (0.01–0.03)	−2.1 (−2.3 to −1.89)
55–59 years	165 (76–270)	0.09 (0.04–0.15)	194 (88–320)	0.05 (0.02–0.08)	−2 (−2.16 to −1.85)
60–64 years	325 (151–515)	0.2 (0.09–0.32)	329 (154–536)	0.1 (0.05–0.17)	−2.23 (−2.37 to −2.09)
65–69 years	534 (262–831)	0.43 (0.21–0.67)	584 (275–929)	0.21 (0.1–0.34)	−2.16 (−2.29 to −2.02)
70–74 years	597 (290–932)	0.7 (0.34–1.1)	912 (441–1,430)	0.44 (0.21–0.69)	−1.71 (−1.85 to −1.57)
75–79 years	815 (417–1,245)	1.32 (0.68–2.02)	1,030 (513–1,651)	0.78 (0.39–1.25)	−1.53 (−1.64 to −1.43)
80–84 years	675 (307–1,043)	1.91 (0.87–2.95)	1,088 (495–1757)	1.24 (0.56–2.01)	−1.26 (−1.45 to −1.08)
85–89 years	346 (153–561)	2.29 (1.01–3.71)	780 (336–1,269)	1.71 (0.74–2.77)	−0.53 (−0.84 to −0.22)
90–94 years	110 (48–187)	2.57 (1.13–4.36)	408 (170–696)	2.28 (0.95–3.89)	0.04 (−0.2–0.29)
95 + years	24 (10–40)	2.31 (0.95–3.91)	144 (55–243)	2.64 (1.01–4.47)	0.85 (0.68–1.02)
SDI region					
High-middle SDI	896 (442–1,391)	0.1 (0.05–0.15)	1,429 (680–2,297)	0.07 (0.03–0.12)	−0.9 (−1.1 to −0.7)
High SDI	2,592 (1240–4,085)	0.22 (0.11–0.35)	3,397 (1601–5,448)	0.14 (0.07–0.23)	−1.3 (−1.43 to −1.17)
Low-middle SDI	34 (15–65)	0.01 (0–0.01)	165 (72–286)	0.01 (0.01–0.02)	2.17 (2.09–2.25)
Low SDI	3 (1–7)	0 (0–0)	15 (6–30)	0 (0–0.01)	3.22 (3.07–3.36)
Middle SDI	165 (75–293)	0.02 (0.01–0.03)	575 (265–972)	0.02 (0.01–0.04)	0.36 (0.19–0.52)

**Table 2:** The number of DALYs cases and the age-standardized DALYs rate attributable to asbestos exposure in 1990 and 2021, and its trends from 1990 to 2021 globally in ovarian cancer.

Characteristics	1990		2021		1990–2021
	Number of DALYs cases (95% UI)	The age-standardized DALYs rate/100000 (95% UI)	Number of DALYs cases (95% UI)	The age-standardized DALYs rate/100000 (95% UI)	EAPC (95% CI)
Global	71,726 (34302–112,279)	1.93 (0.92–3.03)	98,122 (47184–156,828)	1.15 (0.56–1.85)	−1.57 (−1.69 to −1.46)
Age					
20–24 years	0 (0–2)	0 (0–0)	1 (0–3)	0 (0–0)	−0.66 (−1.61–0.29)
25–29 years	8 (2–18)	0 (0–0)	9 (2–22)	0 (0–0)	−1.38 (−2.05 to −0.7)
30–34 years	14 (4–33)	0 (0–0.01)	19 (5–44)	0 (0–0.01)	−1.04 (−1.56 to −0.53)
35–39 years	131 (57–232)	0.04 (0.02–0.07)	152 (65–280)	0.03 (0.01–0.05)	−1.69 (−1.94 to −1.44)
40–44 years	380 (168–663)	0.13 (0.06–0.23)	495 (212–900)	0.1 (0.04–0.18)	−1.27 (−1.44 to −1.1)
45–49 years	904 (397–1,564)	0.39 (0.17–0.67)	1,110 (498–1984)	0.23 (0.11–0.42)	−1.97 (−2.26 to −1.69)
50–54 years	2,846 (1293–4,622)	1.34 (0.61–2.17)	3,128 (1389–5,276)	0.7 (0.31–1.19)	−2.09 (−2.3 to −1.88)
55–59 years	5,649 (2615–9,243)	3.05 (1.41–4.99)	6,700 (3043–11,013)	1.69 (0.77–2.78)	−1.99 (−2.15 to −1.84)
60–64 years	9,601 (4463–15,230)	5.98 (2.78–9.48)	9,776 (4563–15,935)	3.05 (1.43–4.98)	−2.22 (−2.36 to −2.08)
65–69 years	13,330 (6535–20,847)	10.78 (5.29–16.87)	14,600 (6855–23,277)	5.29 (2.49–8.44)	−2.14 (−2.28 to −2.01)
70–74 years	12,235 (5940–19,186)	14.45 (7.02–22.66)	18,696 (9006–29,388)	9.08 (4.38–14.28)	−1.71 (−1.85 to −1.57)
75–79 years	13,279 (6796–20,384)	21.57 (11.04–33.12)	16,819 (8402–26,895)	12.75 (6.37–20.39)	−1.54 (−1.64 to −1.43)
80–84 years	8,665 (3945–13,417)	24.49 (11.15–37.93)	13,945 (6348–22,543)	15.92 (7.25–25.74)	−1.27 (−1.46 to −1.09)
85–89 years	3,516 (1558–5,688)	23.27 (10.31–37.64)	7,895 (3405–12,817)	17.27 (7.45–28.03)	−0.54 (−0.85 to −0.23)
90–94 years	972 (426–1,647)	22.67 (9.95–38.43)	3,599 (1492–6,120)	20.12 (8.34–34.21)	0.04 (−0.2–0.29)
95 + years	195 (80–330)	19.14 (7.9–32.37)	1,177 (451–1987)	21.6 (8.27–36.45)	0.81 (0.64–0.98)
SDI region					
High-middle SDI	19,583 (9564–30,615)	1.97 (0.96–3.07)	27,537 (13178–44,881)	1.36 (0.65–2.22)	−1.25 (−1.43 to −1.06)
High SDI	47,576 (22826–74,730)	4.15 (1.98–6.53)	54,600 (26157–86,834)	2.41 (1.16–3.83)	−1.61 (−1.73 to −1.5)
Low-middle SDI	732 (309–1,449)	0.13 (0.06–0.26)	3,460 (1491–5,975)	0.26 (0.11–0.44)	2.14 (2.05–2.24)
Low SDI	63 (15–167)	0.03 (0.01–0.07)	330 (121–693)	0.07 (0.03–0.15)	3.05 (2.92–3.18)
Middle SDI	3,700 (1660–6,706)	0.39 (0.18–0.7)	12,076 (5470–21,056)	0.46 (0.21–0.79)	0.38 (0.24–0.52)

**Figure 3:**
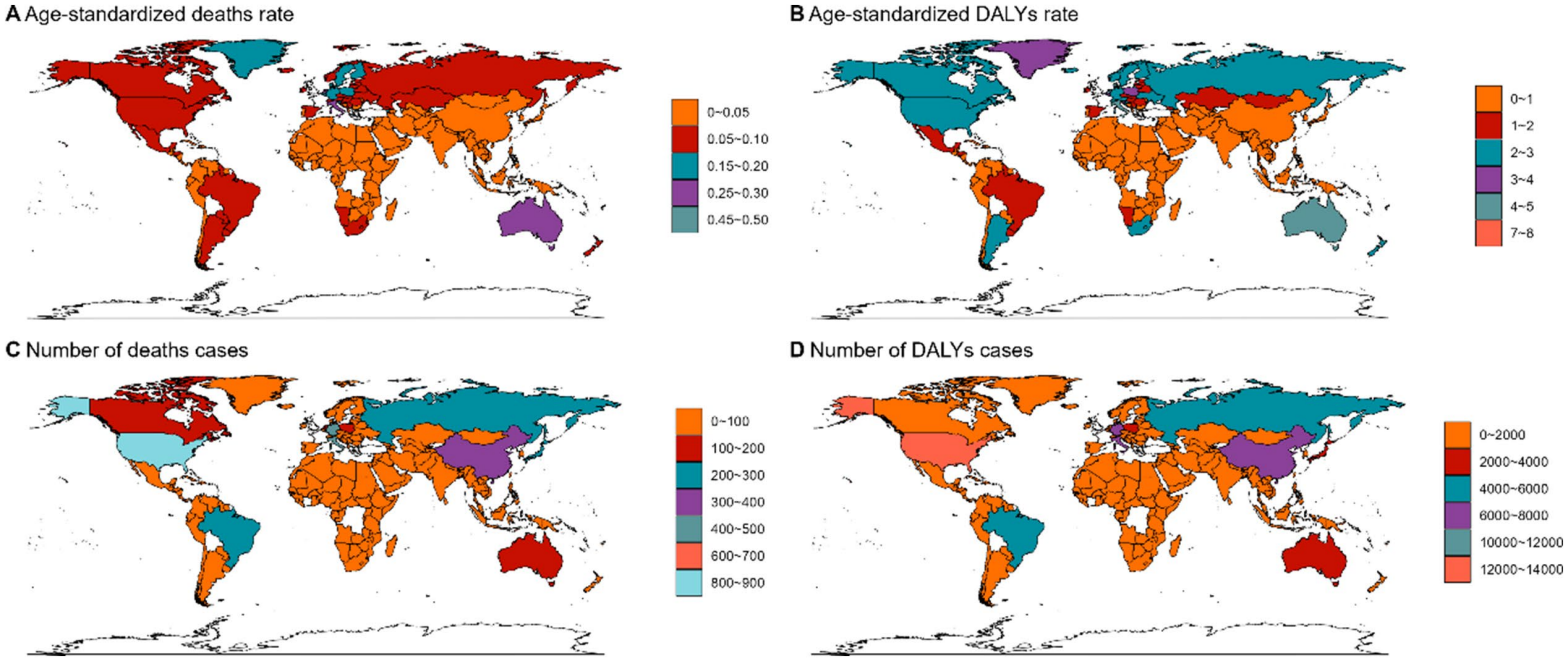
The age standardized deaths rate **(A)**, age standardized DALYs rate **(B)**, number of deaths cases **(C)** and number of DALYs cases **(D)** of OC attributed to asbestos in different regions and countries around the world.

In 2021, regions with a high sociodemographic index (SDI) recorded the highest number of ovarian cancer-related deaths (3,397) and DALYs (54,600) (Figure 1C, 3). Age-stratified analyses for 2021 demonstrated that asbestos-associated mortality and disability rates increased progressively with age, peaking most prominently in individuals aged over 70 years (Figure 1D). Death counts and DALY cases followed a unimodal distribution, with maxima observed in the 80–84 and 70–74 age cohorts (Figure 1D). Notably, while Eastern Sub-Saharan Africa exhibited significant increases in both mortality and DALYs, regions such as Eastern Europe experienced pronounced declines (Figure 1E). The age standard rates of Deaths and DALYs exhibit a growth trend in the next 20 years (Figure 1F).

### Asbestos exposure and ovarian cancer: epidemiological contributions to global health burden

ASMR and ASDR associated with asbestos-related ovarian cancer declined from 0.11 (95% UI: 0.05–0.17) and 1.93 (95% UI: 0.92–3.03) per 100,000 population in 1990 to 0.07 (95% UI: 0.03–0.11) and 1.15 (95% UI: 0.56–1.85) per 100,000 in 2021, respectively (Figure 1A; Tables 1, 2).

Between 1990 and 2021, substantial reductions in ASMR and ASDR were documented in high and high-middle SDI regions, while minimal changes occurred in other geographic areas (Figures 2A,B). Overall, age-stratified analyses revealed consistent downward trends for both metrics across demographic subgroups.

While the absolute number of asbestos-attributable OC deaths rise from 1990 and 2021, the age-standardized rates of Deaths and DALYs decline in different age group (Figure 2C).

### Projection of incidence and mortality trends in ovarian cancer associated with asbestos exposure

A backpropagation neural network model, optimized for goodness-of-fit, was used to forecast ovarian cancer incidence and mortality trends associated with asbestos exposure from 2022 to 2046. Predictive analysis indicates that while both rates are projected to continue rising over the next 25 years, this rise is expected to decelerate. Our estimates suggest that asbestos-linked ovarian cancer will account for 6,167 fatalities and 123,014 DALYs during this period (Figure 1F).

## Discussion

Over the past three decades, ovarian cancer (OC) has experienced evolving epidemiological patterns driven by environmental and demographic shifts. This study provides a comprehensive global assessment of these trends, revealing a critical divergence: while the absolute number of asbestos-related OC deaths and DALYs has risen by 51.2 and 40.4% respectively, the age-standardized mortality rate (ASMR) and DALY rate have significantly declined. This contrast highlights the shifting dynamics of disease burden versus risk exposure.

The observed decline in ASMR and DALY rates likely reflects the global reduction in asbestos utilization since the late 20th century. As stricter occupational health regulations and asbestos bans have been implemented, the exposure intensity in female cohorts has progressively decreased (13, 14). However, the paradoxical rise in absolute death counts is primarily driven by global population aging and growth. Since OC mortality is disproportionately concentrated in older adults (peaking in the 80–84 age group), the demographic expansion of this high-risk cohort outweighs the benefits of reduced risk rates, leading to a heavier total societal burden.

These findings align with and extend existing literature. Consistent with Stoppa G et al., who emphasized the need for stratified analysis in linking asbestos to OC (15), our age-stratified results confirm that while the independent risk contribution of asbestos is diminishing, the legacy effect in older populations remains significant. Unlike studies focusing solely on incidence, our analysis integrates DALYs to capture the full health impact, corroborating recent GBD analyses that identify demographic shifts as the primary driver of modern cancer burden (1).

From a public health perspective, these results suggest a shift in intervention focus. While primary prevention through asbestos control has proven effective in lowering rates, the rising absolute burden necessitates strengthened secondary prevention and clinical care for the aging population. Future health policies should prioritize early screening and monitoring for older adults with historical occupational exposure, even as new occupational risks decline.

Ovarian cancer (OC) mortality demonstrates a disproportionate concentration among older populations. Data indicate significantly increased OC incidence within the 65–84 age group. However, asbestos-attributable mortality and DALYs exhibit declining trends, potentially linked to the progressive reduction in asbestos product use since the late 20th century and the consequent decrease in exposed female cohorts (13). Despite this trend, preventive health measures for older adults should be strengthened to address long-latency health consequences from historical asbestos exposure. Future research priorities should include investigation of additional environmental risk factors—such as talcum powder and cigarette exposure—with pathogenic mechanisms similar to asbestos (16).

Our predictive analysis reveals a divergence between absolute burden and epidemiological rates. While the absolute number of asbestos-attributable ovarian cancer deaths and DALYs is projected to rise between 2021 and 2046—driven primarily by global population growth and aging—the age-standardized mortality and incidence rates are anticipated to decline. Despite the decline in standardized rates, the complexity of etiology requires further scrutiny. Our findings suggest that asbestos’ role as an independent risk factor is waning relative to other exposures; However, its interaction with other factors remains relevant. The etiology of ovarian cancer should be conceptualized as multifactorial, involving potential synergistic effects between decreasing environmental exposures (e.g., asbestos) and increasing endogenous factors associated with aging. Future research should prioritize: (1) strengthening epidemiological evidence for established environmental risk factors, and (2) expanding investigations to include additional environment-related variables for systematic validation.

This study is constrained by several methodological limitations that warrant careful interpretation. First, the reliance on retrospective GBD datasets may introduce estimation bias, as historical diagnostic records often lack granular baseline demographic and clinical covariates. Specifically, the potential misclassification of peritoneal mesothelioma as ovarian cancer in earlier records could lead to an overestimation of the asbestos-attributable fraction in historical data. Second, our predictive framework relies on static assumptions regarding unmodeled confounding factors throughout the 25-year projection window. Consequently, these predictive outcomes should be interpreted as reflecting epidemiological “trends” rather than absolute “certainty.” Finally, the inherent epidemiological profile of ovarian cancer—characterized by lower population-level incidence compared to other malignancies—may result in wider uncertainty intervals in regional estimates, particularly in low-SDI regions with less robust registry systems.

## Conclusion

In conclusion, the significance of asbestos exposure as an independent risk factor for ovarian cancer is waning. Future research should: (1) incorporate supplementary environmental exposure metrics in population cohort studies, (2) strengthen mechanistic investigations to substantiate asbestos’s etiological role, and (3) evaluate synergistic effects among multiple risk factors.

Despite declining attributable fractions, the overall incidence of ovarian cancer continues to rise. This necessitates correlating disease burden metrics with population-level interventions, implementing targeted prevention strategies, and developing translational frameworks to mitigate progression trajectories.

## Data Availability

The original contributions presented in the study are included in the article/supplementary material, further inquiries can be directed to the corresponding author.

## References

[1] Li T Zhang H Lian M He Q Lv M Zhai L . Global status and attributable risk factors of breast, cervical, ovarian, and uterine cancers from 1990 to 2021. J Hematol Oncol. (2025) 18:5. doi: 10.1186/s13045-025-01660-y, 39794860 PMC11721161

[2] Huang J Pang WS Fung YC Mak FY Chan SC Liu X . Global burden, risk factors, and temporal trends of ureteral cancer: a comprehensive analysis of cancer registries. BMC Med. (2024) 22:264. doi: 10.1186/s12916-024-03485-x 38915094 PMC11197334

[3] Ness RB Cottreau C. Possible role of ovarian epithelial inflammation in ovarian cancer. J Natl Cancer Inst. (1999) 91:1459–67. doi: 10.1093/jnci/91.17.1459, 10469746

[4] Pietrofesa RA Velalopoulou A Albelda SM Christofidou-Solomidou M. Asbestos induces oxidative stress and activation of Nrf2 Signaling in murine macrophages: chemopreventive role of the synthetic lignan secoisolariciresinol diglucoside (LGM2605). Int J Mol Sci. (2016) 17:322. doi: 10.3390/ijms17030322, 26938529 PMC4813184

[5] Hillegass JM Miller JM MacPherson MB Westbom CM Sayan M Thompson JK . Asbestos and erionite prime and activate the NLRP3 inflammasome that stimulates autocrine cytokine release in human mesothelial cells. Part Fibre Toxicol. (2013) 10:39. doi: 10.1186/1743-8977-10-39, 23937860 PMC3751315

[6] Cox LA Jr. Dose-response modeling of NLRP3 inflammasome-mediated diseases: asbestos, lung cancer, and malignant mesothelioma as examples. Crit Rev Toxicol. (2019) 49:614–35. doi: 10.1080/10408444.2019.1692779, 31905042

[7] Di Mauro G Frontini F Torreggiani E Iaquinta MR Caselli A Mazziotta C . Epigenetic investigation into circulating microRNA 197-3p in sera from patients affected by malignant pleural mesothelioma and workers ex-exposed to asbestos. Sci Rep. (2023) 13:6501. doi: 10.1038/s41598-023-33116-z, 37081052 PMC10119131

[8] Nuvoli B Galati R. Cyclooxygenase-2, epidermal growth factor receptor, and aromatase signaling in inflammation and mesothelioma. Mol Cancer Ther. (2013) 12:844–52. doi: 10.1158/1535-7163.MCT-12-1103, 23729401

[9] GBD 2021 Global AMD Collaborators. Global burden of vision impairment due to age-related macular degeneration, 1990-2021, with forecasts to 2050: a systematic analysis for the global burden of disease study 2021. Lancet Glob Health. (2025) 13:e1175–90. doi: 10.1016/S2214-109X(25)00143-3 40580986 PMC12208786

[10] GBD 2023 Vaccine Coverage Collaborators. Global, regional, and national trends in routine childhood vaccination coverage from 1980 to 2023 with forecasts to 2030: a systematic analysis for the global burden of disease study 2023. Lancet. (2025) 406:235–60. doi: 10.1016/S0140-6736(25)01037-2 40578370 PMC12338332

[11] Deng L Du C Liu L Wang Y Gu H Armstrong DG . Forecasting the global burden of peripheral artery disease from 2021 to 2050: a population-based study. Research. (2025) 8:0702. doi: 10.34133/research.0702 40599301 PMC12209533

[12] Collaborators GBDRF. Global burden and strength of evidence for 88 risk factors in 204 countries and 811 subnational locations, 1990-2021: a systematic analysis for the global burden of disease study 2021. Lancet. (2024) 403:2162–203. doi: 10.1016/S0140-6736(24)00933-4 38762324 PMC11120204

[13] Henley SJ Peipins LA Rim SH Larson TC Miller JW. Geographic co-occurrence of mesothelioma and ovarian Cancer incidence. J Womens Health (Larchmt). (2020) 29:111–8. doi: 10.1089/jwh.2019.7752, 31314677 PMC6962528

[14] Wang Y Wang Z Zhang Z Wang H Peng J Hong L. Burden of ovarian cancer in China from 1990 to 2030: a systematic analysis and comparison with the global level. Front Public Health. (2023) 11:1136596. doi: 10.3389/fpubh.2023.1136596, 36860393 PMC9969192

[15] Stoppa G Mensi C Fazzo L Minelli G Manno V Marinaccio A . Ovarian cancer deaths attributable to asbestos exposure in Lombardy (Italy) in 2000-2018. Occup Environ Med. (2024) 81:359–65. doi: 10.1136/oemed-2023-109342, 38981677 PMC11347218

[16] Reid BM Permuth JB Sellers TA. Epidemiology of ovarian cancer: a review. Cancer Biol Med. (2017) 14:9–32. doi: 10.20892/j.issn.2095-3941.2016.0084, 28443200 PMC5365187

