# Correction: Global trend analysis and risk evolution of asbestos-related ovarian cancer: a population-based study and future prediction (1990–2021)

**DOI:** 10.3389/fpubh.2026.1785041

**Published:** 2026-02-02

**Authors:** Xiaolong Li, Jiwei Li, Dongyong Shan, Ming Zhou, Mengna Li, Yinghua Li, Man Xia, Hongyu Deng

**Affiliations:** 1 Department of Clinical Laboratory, Hunan Key Laboratory of Oncotarget Gene, Hunan Cancer Hospital and The Affiliated Cancer Hospital of Xiangya School of Medicine, Central South University, Changsha, China; 2 Department of Oncology, The Second Xiangya Hospital, Central South University, Changsha, China; 3 Department of Gynecological Oncology, Hunan Cancer Hospital and the Affiliated Cancer Hospital of Xiangya School of Medicine, Central South University, Changsha, China

**Keywords:** asbestos, epidemiology, GBD, global disease burden 2021, ovarian cancer

Affiliation 2. Department of Oncology, The Second Xiangya Hospital, Central South University, Changsha, China was omitted for authors Xiaolong Li, Jiwei Li, and Dongyong Shan. This affiliation has now been added for authors Xiaolong Li, Jiwei Li, and Dongyong Shan.

Affiliation 1. Department of Clinical Laboratory, Hunan Key Laboratory of Oncotarget Gene, Hunan Cancer Hospital and the Affiliated Cancer Hospital of Xiangya School of Medicine, Central South University, Changsha, China was omitted for authors Ming Zhou, Mengna Li, and Hongyu Deng. This affiliation has now been added for authors Ming Zhou, Mengna Li, and Hongyu Deng.

Authors Jiwei Li and Dongyong Shan were erroneously assigned to affiliation 1. Department of Clinical Laboratory, Hunan Key Laboratory of Oncotarget Gene, Hunan Cancer Hospital and the Affiliated Cancer Hospital of Xiangya School of Medicine, Central South University, Changsha, China. This affiliation has now been removed for authors Jiwei Li, and Dongyong Shan.

Authors Ming Zhou, Mengna Li, and Hongyu Deng were erroneously assigned to affiliation 2. Department of Oncology, The Second Xiangya Hospital, Central South University, Changsha, China. This affiliation has now been removed for authors Ming Zhou, Mengna Li, and Hongyu Deng.

Author Mengna Li was erroneously spelled as Li Mengna.

The author contributions statement was erroneously given as LM: Formal analysis, Writing – original draft, Project administration. The correct author contributions statement is ML: Formal analysis, Writing – original draft, Project administration.

The original version of this article has been updated.

